# Retraction Note: Antibacterial Activity of Green Synthesized Silver Nanoparticles Using Lawsonia Inermis Against Common Pathogens from Urinary Tract Infection

**DOI:** 10.1007/s12010-026-05870-z

**Published:** 2026-08-11

**Authors:** Ahmed Said, Mohammed Abu-Elghait, Hossam M. Atta, Salem S. Salem

**Affiliations:** Botany and Microbiology Department, Faculty of Science, Al-Azhar University, Nasr City, Cairo, 11884 Egypt

**Retraction Note: Applied Biochemistry and Biotechnology (2023) 196:85–98**

**https://doi.org/10.1007/s12010-023-04482-1**

The Editors-in-Chief have retracted this article after an investigation found that the peer review process had not been conducted in accordance with the Journal’s editorial policies. Additionally, concerns were raised about the inclusion of a high number of authors’ citations. The authors were unable to provide a satisfactory response to raised concerns.

Salem S. Salem disagrees with this retraction. Hossam M. Atta did not respond to any correspondence from the publisher regarding this retraction. The Publisher was unable to find current email addresses of Ahmed Said and Mohammed Abu-Elghait.

